# Spatial distribution and determinants of children ever born among reproductive age women in Ethiopia: spatial and multilevel analysis of 2019 mini Ethiopian demographic health survey

**DOI:** 10.3389/frph.2025.1389932

**Published:** 2025-02-10

**Authors:** Ahmed Fentaw Ahmed, Bezawit Adane, Tilahun Degu Tsega, Mekides Nigusu, Kalaab Esubalew Sharew, Abebaw Molla, Mulugeta Tesfa

**Affiliations:** ^1^ Department of Public Health, College of Medicine and Health Sciences, Injibara University, Injibara, Ethiopia; ^2^ Department of Nursing, College of Medicine and Health Sciences, Injibara University, Injibara, Ethiopia; ^3^ Department of Medicine, College of Medicine and Health Sciences, Injibara University, Injibara, Ethiopia; ^4^ Department of Public Health, College of Medicine and Health Sciences, Debre Markos University, Debremarkos, Ethiopia

**Keywords:** children ever born, fertility status, spatial analysis, multi-level analysis, Ethiopia

## Abstract

**Background:**

Understanding population dynamics is essential since the number of children ever born (CEB) affects the growth, composition, and structure of a nation's population. The number of CEB has increased significantly, contributing to the world's rapid population growth. The spatial distribution of CEB in Ethiopia lacks recent information. Therefore, this study aimed to assess spatial distribution, and associated factors of CEB among reproductive age women in Ethiopia.

**Method:**

Mini Ethiopian Demographic and Health Survey (MEDHS) 2019 data were used in this study. The study comprised 5527 (weighted) women's between the ages of 15 and 49. STATA and Aeronautical Reconnaissance Coverage Geographic Information System (ArcGIS) 10.8 software was used. The primary outcome, CEB, was categorized as “low” if fewer than five children were born and “high” if five or more children were born. Global and local Moran's Index methods were used to assess the extent of clustering. Multi-level (two-level) logistic regression analysis was used and variables with a *P* value less than 0.05 were considered statistical significance. Adjusted odds ratio AOR) with a 95% confidence interval (CI) was used to show the strength and direction of the association respectively.

**Results:**

High number of CEB in Ethiopia was 37.46%, 95% CI (0.39–0.56) and it was spatially clustered (Moran's index = 0.59 *P* value <0.0001). Significant hotspots of high CEB were found in the Eastern Somali, Hadiya, Sidama, and Welayta zones. From individual-Level variables: women who were married [AOR = 3.23, 95% CI (1.48, 6.62)] were positively associated with high number of CEB. Whereas, women who were primary educated [AOR = 0.18, 95% CI (0.12, 0.27)], women who were secondary educated [AOR = 0.0.05, 95% CI (0.02, 0.13)], women's whose age at first birth after 20 year [AOR = 0.38, 95% CI (0.27, 0.51)] and women's who were using contraceptive [AOR = 0.59, 95% CI (0.44–0.78) were negatively associated with high number of CEB. From community level variables: a community with high proportion of contraceptive non user [AOR = 1.38, 95% CI (1.94–2.04)] were positively associated with high number of CEB.

**Conclusion:**

Both individual and community-level factors were significantly linked to a high number of children born. The government is advised to prioritize interventions that promote women's education, delay first births, and provide access to a range of contraceptive options, ensuring informed, voluntary choices. Empowering women to exercise reproductive autonomy, free from coercion, is key to influencing fertility outcomes effectively.

## Introduction

Understanding population dynamics is essential since the number of children ever-born (CEB) affects the growth, composition, and structure of a nation's population. The average number of children born alive to women within a given age range is referred to as “ children ever-born.” It includes all live births among married women of reproductive age, regardless of whether the offspring are still living or have passed away (1). The number of CEB has increased significantly, contributing to the world's rapid population growth. In 2020, the world population grew at a rate of less than 1 percent annually for the first time since 1950. The percentage of the population in working age (between 25 and 64 years old) has increased significantly in several sub-Saharan African countries as well as in some Asian, Latin American, and Caribbean regions. The decrease in fertility rates is the main cause of this change. This shift in the distribution of ages offers a finite chance for increased economic growth per individual, or the “demographic dividend” (2).

In 2020, the global total fertility rate (TFR) was projected to be 2.4 births per woman, which is little higher than the replacement level of 2.1 (3). With 4.1 births per woman in 2020, Africa has the highest TFR of any continent (3, 4). Ethiopia had a TFR of 4.0 births per woman in 2020, which was higher than the global average but lower than the sub-Saharan African average (5). Ethiopia's total fertility rate (TFR) has drastically decreased from 6.8 births per woman in 1990 as a result of a number of reasons, including more accessibility to family planning, healthcare, and education (6). While TFR provides a snapshot of fertility levels, it is a projected estimate and does not account for the actual number of children women have over their lifetimes, which is captured by the CEB. Therefore, examining the determinants of CEB offers a more comprehensive understanding of fertility patterns and individual reproductive behavior.

The number of children a woman has can serve as an indicator of her rights and autonomy, especially regarding reproductive rights and gender equality. Having the power to determine the number and timing of children is a key element of reproductive rights. When women have control over these decisions, it typically reflects greater autonomy and access to healthcare services, such as contraception and safe abortion (7). CEB can serve as an indicator of a woman's right to choose because it reflects her autonomy over reproductive decisions, such as the number and timing of her children. When women are empowered with reproductive rights—access to contraception, family planning, and healthcare services—they are more likely to exercise control over these decisions. In contexts where reproductive rights are limited, high CEB may indicate that women lack the ability to make informed choices due to cultural, social, or economic barriers (8).

As evidenced by various studies, several factors are associated with the number of CEB. Age is a significant factor, with older women more likely to have higher CEB due to extended exposure to childbearing (9, 10). Literacy is also closely linked to higher CEB; women with lower educational attainment often have less access to family planning resources and information, leading to higher fertility rates (11). Marital status plays a crucial role, as married women typically have more children than their unmarried counterparts due to societal norms and expectations (12). Religion (1, 13–15), sex of household head (16), wealth index (1, 8, 10, 11, 15, 17–21), age at first marriage (11–13, 15, 20–23), exposure to mass media (12, 15, 22), have dead child can impact CEB, as families may have additional children to replace those lost (11, 21), sex of child can also play a role, with cultural preferences influencing family size (15). Contraceptive utilization (11, 19, 20, 21, 24) is another significant factor affecting CEB.Community-level variables such as community level education (13), community-level of wealth status (13), community-level media exposure (13), community contraceptive utilization (25), and residences (9, 10, 13, 15, 17, 18, 20, 21, 22, 26) have also been found to be important determinants of CEB. Particularly in low- and middle-income countries, including Sub-Saharan Africa, such as Ethiopia. These factors highlight the complexity of fertility determinants across different regions.

This study seeks to address the persistent issue of a high number of CEB in Ethiopia, where both individual and community-level factors play a significant role. Existing research has largely focused on the total fertility rate (TFR) and individual-level determinants, leaving gaps in understanding the spatial distribution and community-level influences on cumulative fertility. By choosing “children ever born” (CEB) over TFR, this research provides a more comprehensive reflection of women's lifetime fertility, offering insights into shifts in fertility behavior over time. Additionally, the lack of recent data on the spatial variation of CEB in Ethiopia further justifies this study. Through spatial and multilevel analysis, this research will explore geographic disparities in fertility, identifying regional hotspots and clusters. This research aims to provide data that informs policymakers about the necessary conditions to enhance women's rights, such as improving education, access to comprehensive reproductive healthcare, and availability of contraceptive options. By focusing on enabling women to make informed choices regarding their fertility, this study ultimately seeks to support the development of targeted public health interventions and guide resource allocation to high-fertility areas, fostering an environment where women can exercise their reproductive autonomy.

## Methods and materials

### Study area and period

The research was undertaken in Ethiopia, the second most populous country Africa, situated in the horn of Africa, comprising nine regional states and two city administrations. The 2019 MEDHS marked the second MEDHS and the fifth national-level demographic and health survey. This study was conducted between November 10, 2023, and January 30, 2024 and this survey utilized a nationally representative sample to examine spatial patterns and determinants of CEB at the national, regional, and zonal levels. The survey interviewed 8,855 women of reproductive age (15–49) from a nationally representative sample of 8,663 households.

### Study design and population

A population-based cross-sectional study was conducted using the dataset from the 2019 MEDHS. The study included all reproductive age women who had given birth in their lifetime within the randomly selected 305 enumeration areas (EAs) in Ethiopia.

### Data source and extraction

We obtained the 2019 MEDHS datasets from the DHS program website (https://www.dhsprogram.com/data/dataset_admin), where they are publicly accessible to registered users. Following a thorough review and comprehension of the EDHS data structure and various dataset types, we specifically chose the recommended dataset type for CEB. Subsequently, data pertaining to CEB, as well as potential independent variables encompassing individual and community-level characteristics, were extracted in accordance with the study requirements.

### Sample size and sampling procedure

**Figure 1:**
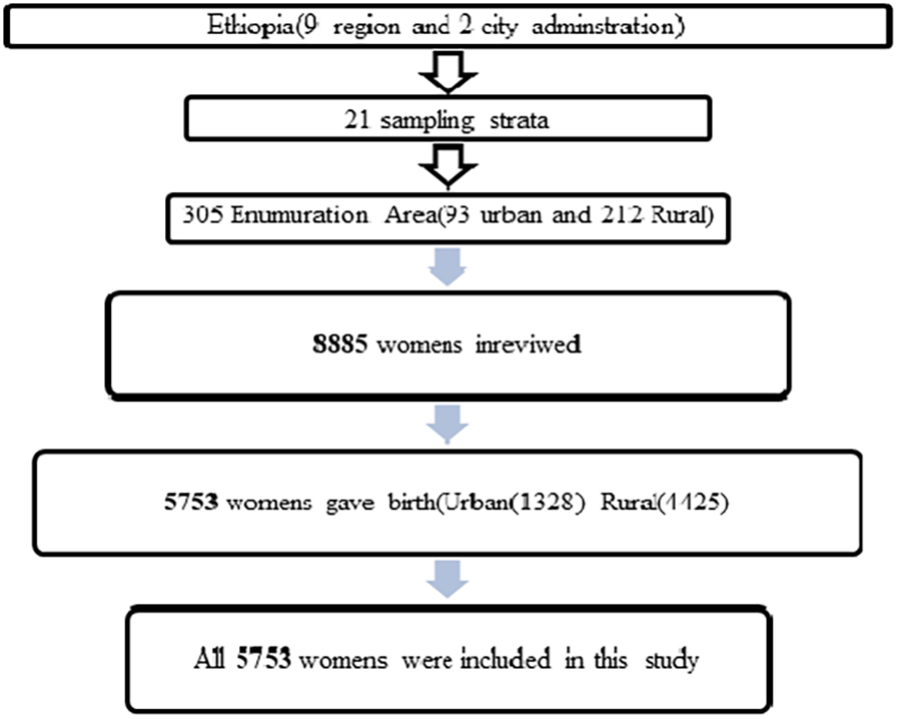
Sampling procedure for spatial distribution and associated factors of Children Ever Born in Ethiopia.

The sampling frame used for the 2019 MEDHS is a frame of all census Enumeration Areas (EAs) created for the upcoming 2019 Ethiopia Population and Housing Census (PHC), which was conducted by the Central Statistical Agency (CSA). The 2019 EDHS used a stratified, two-stage cluster sampling design using the enumeration areas 305 (EAs) as the primary sampling unit and households as the secondary sampling unit. The survey collected information from a nationally representative sample of 5,753 women's in reproductive age (15–49) who gave birth before the survey (Figure 1).

### Variable measurement

The dependent variable in this research is classified as “low” if the number of CEB is less than five and “high” if CEB is five or more, specifically among currently married women. This cut-off is chosen based on evidence suggesting that having five or more children is linked to increased maternal and child health risks, including higher rates of maternal morbidity and mortality (13). Additionally, it reflects broader socio-economic factors influencing reproductive choices, making it a significant threshold for understanding fertility patterns in this context (27). A woman will receive a code of 0 (indicating low CEB) if she has given birth to fewer than five children, and a code of 1 (indicating high CEB) if she has given birth to five or more children (13).

#### Independent variables

The individual explanatory variables to be studied as determinants of children ever born status of married women are: age in 5-year groups, literacy, marital status, educational status of women's, religion, sex of household head, wealth index, age at first marriage, exposure to mass media, have dead child, sex of child, and contraceptive.

Community-level variables were derived by aggregating individual-level variables, given that the Ethiopian Demographic and Health Survey (EDHS) did not directly collect community-level variables, apart from residence and region. The aggregation process involved calculating the proportions of each variable's subcategories within a given cluster. Due to the non-normal distribution of the aggregated values for all variables, they were subsequently grouped based on their respective national median values. To classify each EA as having high or low proportion, we compare the EA-level median to the national median: High proportion: If the EA-level median is greater than or equal to the national median, the community is classified as having high proportion. Low proportion: If the EA-level median is less than the national median, the community is classified as having low proportion.

Community-level of education, community-level of wealth status, community-level media exposure, community family disruption, community literacy, community contraceptive utilization, region and residences were considered as community level variables.

### Operational definition

• Contraceptive use refers specifically to whether a woman is currently using any form of contraception, without distinguishing between modern or traditional methods, unless otherwise specified (28).
• Community Media Exposure: The availability of TV and radio among respondents in a cluster is categorized as low if less than 50% of households possess these media devices and high if 50% or more of households have access to TV or radio (29).
• Community family disruption: refers to the proportion of women living in households led by females. Communities were categorized as having a high level of family disruption if this percentage surpassed the median value; conversely, those below the median were considered to have a low level of family disruption (30).
• Community contraceptive utilization refers to the proportion of reproductive-age women who use contraceptives within a given community. If this percentage exceeded the median value, the community was classified as having a high level of contraceptive utilization. On the other hand, communities with a percentage below the median were categorized as having a low level of contraceptive utilization (29).
• Community level educational status: Community educational status was determined by the proportion of women of reproductive age who had education beyond primary school. If the proportion exceeded the median value, the community was classified as having a high proportion of education; otherwise, it was categorized as having a low proportion of community-level educational status (30).
• Community level poverty: he community's poverty status was measured by the percentage of individuals identified as poor (including both the poorest and poor categories) within each cluster. If the proportion was above the median, the community was classified as having a high proportion of poverty; otherwise, it was categorized as having a low proportion of community wealth status (29). Wealth index: The wealth index serves as an indicator of household economic status and is calculated based on household assets. Households are categorized into five groups: poorest, poorer, middle, richer, and richest (31).

## Data processing and analysis

### Multilevel and descriptive analysis

Before proceeding with the data analysis, several tasks were performed, including data cleaning, recoding, labeling, and weighting, to ensure consistency and address missing values. Sampling weights were applied using the individual women's sample weight variable, following DHS recommendations. Specifically, the weight variable (v005) was divided by 1,000,000 to adjust for variations in selection probability. This ensures the data are representative of the broader population by accounting for unequal probabilities of selection in the sampling process. Descriptive analysis, such as frequencies and percentages, was conducted using STATA 17, presenting the results through tables, graphs, and textual descriptions to characterize the participants. Sample weights were applied, and multilevel analysis was carried out after verifying the presence of a significant Intra-cluster Correlation (ICC). Given the hierarchical nature of DHS data, with individuals (level 1) nested within communities (level 2), a two-level mixed effects logistic regression model was employed to estimate the independent effects of the explanatory variables.

The measures of variation (random effects) were reported using Intra-cluster correlation ($\mathrm{ICC}=\frac{\mathrm{VA}}{\mathrm{VA}+3.29}*100$), Median Odds Ratio $\mathrm{MOR}(=\;e^{0.95}\surd \mathrm{VA})$ and proportional change in variance ($\mathrm{PCV}=\frac{\mathrm{VNull}-\mathrm{VA}}{\mathrm{VNull}}*100$). The Intra-cluster Correlation (ICC) was utilized to elucidate cluster variation, indicating the extent to which community characteristics contribute to the variability in the number of CEB for women's. A higher ICC suggests that community characteristics play a more significant role in understanding individual variation. Meanwhile, the Median Odds Ratio (MOR) is a measure of unexplained cluster heterogeneity, representing the median odds of the odds ratio between the area with the highest risk and the one with the lowest risk when randomly selecting two areas.

The analysis involved a four-step process: Model 1 (empty model or null model without explanatory variables), Model 2 (adjusted for individual-level factors only), Model 3 (adjusted for community-level factors only), and Model 4 (adjusted for both individual and community-level factors). Initially, a bi-variable multi-level logistic regression was conducted, and variables with a *p*-value less than 0.25 were chosen for the final model. Multi-collinearity among independent variables was checked, and no issues were identified. The goodness of fit for the adjusted final model, as compared to the preceding models, was assessed using the log-likelihood ratio test. A significant log-likelihood ratio test and a lower Akaike Information Criterion (AIC) were considered indicators of the best-fit model.

#### Spatial analysis

The combined data of CEB counts and proportions of CEB aggregated at community level were linked to geographic coordinates using the cluster identification code. The Global Moran's I statistic (Moran's I) was employed to assess whether the observed pattern exhibited clustering, dispersion, or randomness across the study areas. Furthermore Hot spot analysis using the Getis-Ord Gi* statistic was conducted to assess the variation in spatial autocorrelation across the study location. The Gi* statistic was calculated for each area to determine the presence and strength of clustering. Statistical significance of these clusters were determined using Z-scores, particularly focusing on CEB.

To predict the CEB levels nationwide with limited sample data points, a spatial interpolation technique, specifically Kriging, was employed.

### Ethical considerations

Ethical approval for this study was secured from the Demographic and Health Surveys (DHS) Program. The study was registered, and a request was submitted, outlining the objectives briefly. Access to the data was granted exclusively for the purpose of this research, and the data could not be shared with other researchers. The required files were downloaded from [https://www.spatialdata.DHSprogram.com].

## Results

### Individual level characteristics of study participants

This analysis incorporated data from a weighted sample of 5,527 women aged 15–49 years who had given birth in their lifetime. Among them, 2,155 (37.46%) had a high number of CEB (5 or number of CEB). The mean age of the respondents was 28.63 ± 0.85 years. A significant portion of the women, 2,962 (53.59%), had no formal education. As per the EDHS 2019 wealth index estimate, 1,321 (23.89%) of the women were categorized as the poorest, while 198 (21.67%) were classified as poorer (Table 1).

**Table 1:** Individual-level characteristics of women of reproductive age by CEB, EDHS 2019 [*n* (weighted) = 5,527].

Variables	Category	Children Ever Born (CEB)	
		Low (<5) CEB (%)	High (≥5) CEB (%)
Age	15–19	263 (7.8)	0
	20–24	1,010 (29.7)	21 (1.0)
	25–29	1,346 (39.6)	410 (19.3)
	30–34	529 (15.3)	674 (31.6)
	35–39	200 (5.9)	607 (28.5)
	40–44	48 (1.4)	319 (15.0)
	45–49	10 (0.3)	99 (4.6)
Marital status	Never in union	236 (6.9)	66 (3.1)
	Married	3,160 (93.1)	2,065 (96.9)
Religion	Orthodox	1,302 (38.3)	558 (26.2)
	Catholic	9 (0.3)	8 (0.4)
	Protestant	909 (26.8)	551 (25.9)
	Muslim	1,133 (33.4)	968 (45.4)
	Traditional	39 (1.1)	40 (1.9)
	Other	4 (0.1)	6 (0.3)
Educational status	No education	1,302 (38.3)	1,660 (77.9)
	Primary	1,515 (44.6)	441 (20.7)
	Secondary	388 (11.4)	27 (1.3)
	Higher	191 (5.6)	3 (0.1)
Literacy	Illiterate	1,868 (55.0)	1,844 (86.5)
	Literate	1,528 (45.0)	287 (13.5)
Head of household	Male	2,905 (85.5)	1,871 (87.8)
	Female	491 (14.5)	260 (12.2)
Wealth index	Poorest	658 (19.4)	663 (31.1)
	Poorer	644 (19.0)	554 (26.0)
	Middle	612 (18.0)	432 (20.3)
	Richer	645 (19.0)	314 (14.7)
	Richest	837 (24.6)	168 (7.9)
Media exposure	Yes	1,378 (40.6)	511 (24.0)
	No	2,018 (59.4)	1,620 (76.0)
Contraceptive utilization	Yes	1,640 (48.3)	612 (28.7)
	No	1,756 (51.7)	1,620 (71.3)
Sex of child	Male	1,763 (51.9)	1,079 (50.6)
	Female	1,633 (48.1)	1,052 (49.4)
Have dead child	Yes	138 (4.1)	138 (6.5)
	No	3,258 (95.9)	1,993 (93.5)
Age at first birth	<20 year	1,865 (54.9)	1,645 (77.2)
	≥20 year	1,531 (45.1)	486 (22.8)

### Community level characteristics of study participants

**Figure 2:**
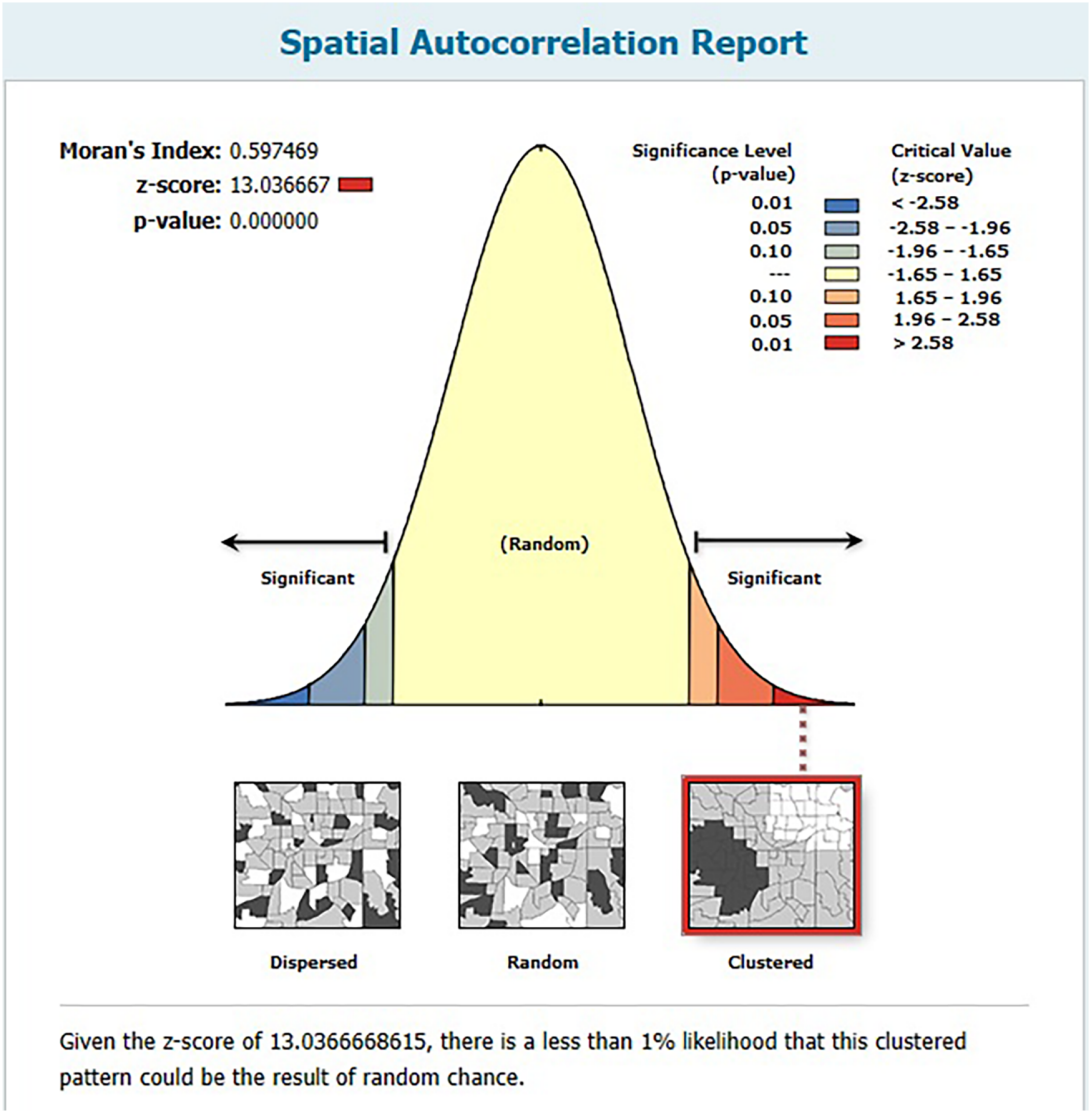
Global spatial autocorrelation of CEB in Ethiopia. 2019 MEDHS.

In this study, a total of 305 clusters were included. When examining the characteristics of these community clusters, 4,160 women (75.27%) were residing in rural areas. Additionally, more than half of the womens, specifically 3,063 (55.4%), belonged to communities with a high proportion of illiteracy. Among the total respondents, 3,065 (55.46%) were situated in communities with a higher proportion of poverty. Notably, nearly half of the womens (48.41%) were from communities characterized by a higher proportion of contraceptive non-use (refer to Table 2). The lowest count of High number of CEB, 5 (0.23), was observed in the Harari region, while the highest count, 970 (45.52), was found in the Oromia region (Figure 2).

**Table 2:** Community-level characteristics of women of reproductive age by CEB, EDHS 2019 [*n* (weighted) = 5,527].

Variables	Category	Children Ever Born (CEB)	
		Low (<5) CEB (%)	High (≥5) CEB (%)
Community education status	Low proportion of bellow secondary	1,735 (51.1)	691 (32.4)
	High proportion of bellow secondary	1,661 (48.9)	1,440 (67.6)
Residence	Urban	1,006 (29.6)	360 (16.9)
	Rural	2,390 (70.4)	1,771 (83.1)
Community level literacy	Low proportion of illiteracy	1,781 (52.4)	683 (32.1)
	High proportion of illiteracy	1,615 (47.6)	1,448 (67.9)
Community wealth status	Low proportion of poor	1,718 (50.6)	744 (34.9)
	high proportion of poor	1,678 (49.4)	1,387 (65.1)
Family disruption	Low proportion	2,037 (60.0)	1,309 (61.4)
	High proportion	1,359 (40.0)	822 (38.6)
Community media exposure	Low proportion of non-exposed	1,634 (48.1)	674 (31.6)
	High proportion of non-exposed	1,762 (51.9)	1,457 (68.4)
Community contraceptive use	Low proportion of non-user	1,991 (58.6)	860 (40.4)
	High proportion of non-user	1,405 (41.4)	1,271 (59.6)
Region	Tigray	270 (7.9)	101 (4.77)
	Afar	55 (1.6)	31 (1.5)
	Amhara	734 (21.6)	316 (14.8)
	Oromia	1,241 (36.5)	970 (45.5)
	Somali	176 (5.2)	232 (10.8)
	Benshangul	40 (1.2)	27 (1.3)
	South Nation Nationalites and Peoples Region (SNNPR)	684 (20.1)	423 (19.9)
	Gambela	17 (0.5)	8 (0.4)
	Harari	11 (0.1)	5 (0.2)
	AA	147 (1.1)	9 (0.4)
	Diredawa	21 (0.6)	9 (0.4)

### Factors associated with Children Ever Born (fixed–effect)

Following adjustments for individual and community-level factors, five variables demonstrated statistical significance in relation to CEB in Ethiopia. At the individual level, these variables included the marital status of the mother, educational attainment, age at first birth, and contraceptive utilization, while at the community level, contraceptive use emerged as significant.

Marital status: Married women were three times more likely to have High number of CEB compared to unmarried women [Adjusted Odds Ratio (AOR) = 3.23%–95% CI: 1.48–6.62]. Educational status: Women with primary education were 82% less likely to have High number of CEB compared to those without education [AOR = 0.18, 95% CI: 0.12–0.27]. Similarly, women with secondary education were 95% less likely to have High number of CEB compared to those without education [AOR = 0.05, 95% CI: 0.02–0.13].

Age at first birth: Women's whose age at first birth was after 20 years were 68% less likely to have High number of CEB compared to those whose age at first birth was before 20 [AOR = 0.38, 95% CI: 0.27–0.51]. Contraceptive utilization: Women's using contraceptives were 41% less likely to have High number of CEB compared to non-users [AOR = 0.59%–95% CI: 0.44–0.78].

Community-level factor - Contraceptive non-use: Women residing in communities with a high proportion of contraceptive non-users were 38% more likely to have High number of CEB compared to those in communities with a low proportion of contraceptive non-users [AOR = 1.38%–95% CI: 1.94–2.04] (refer to Table 3).

**Table 3:** Individual- and community-level factors associated with CEB, EDHS 2019 (*n* = 5,526).

Individual- and community- level characteristics	COR (95% CI)	Model 1 AOR (95% CI)	Model 2 AOR (95% CI)	Model 3 AOR (95% CI)
Education status of women's				
Not educated	1	1		1
Primary	0.17 (0.12–0.24)	018 (012–0.27)		0.18 (0.12–0.27)*
Secondary	0.04 (0.01–0.09)	0.05 (0.02–0.13)		0.05 (0.02–0.13)*
Higher	0.01 (0.01–0.03)	0.12 (0.00–0.08)		0.01 (0.01–0.08)*
Wealth index of household				
Poorest	1	1		1
Poorer	0.82 (0.59–1.16)	0.97 (0.69–1.34)		0.95 (0.67–1.34)
Middle	0.68 (0.45–1.05)	1.0 (0.64–1.57)		0.97 (0.61–1.54)
Richer	0.44 (0.27–0.73)	0.67 (0.37–1.17)		0.66 (0.37–1.19)
Richest	0.23 (0.11–0.46)	0.74 (0.34–1.63)		0.81 (0.31–2.11)
Literacy				
Illiterate	1	1		1
Literate	0.2 (0.14–0.27)	0.86 (0.6–1.24)		0.87 (0.61–1.25)
Age at first birth				
<20	1	1		1
≥20	0.41 (0.31–0.53)	0.34 (0.27–0.50)		0.38 (0.27–0.51)**
Have dead child				
Yes	0.67 (0.43–1,010	0.65 (0.41–1.03)		0.65 (0.41–1.03)
No	1	1		1
Contraceptive utilization				
Yes	0.52 (0.41–0.68)	0.58 (0.44–0.77)		0.59 (0.44–0.78)*
No	1	1		1
Marital status				
Never in union	1	1		1
Married	2.4 (1.36–4.25)	3.03 (1.42–6.44)		3.13 (1.48–6.62)*
Religion				
Orthodox	1	1		1
Catholic	1.55 (0.42–5.6)	1.62 (0.36–7.18)		1.31 (0.28–5.94)
Protestant	1.05 (0.6–1.6)	1.55 (0.97–2.4)		1.32 (0.72–2.41)
Muslim	2.30 (1.57–3.38)	1.74 (1.15–2.64)		1.75 (0.95–3.21)
Traditional	2.23 (1.17–4.25)	2.39 (0.86–6.58)		2.02 (0.74–5.46)
Other	2.530.7 (8–8.15	2.24 (0.84–12.4)		2.51 (0.64–9.87)
Head of household				
Male	1.2 (0.89–1.77)	1.07 (0.70–1.65)		1.03 (0.66–1.63)
Female	1	1		1
Media exposure				
Yes	0.6 (0.44–0.82)	1.05 (0.76–1.45)		1.04 (0.75–1.44)
No	1	1		
Community education status				
Low proportion of bellow secondary	1		1	1
High proportion of bellow secondary	3.16 (2.3–4.3)		1.55 (1.13–2.10)	1.02 (0.70–1.48)
Residence				
Urban	1		1	1
Rural	3.6 (2.26–5.79)		1.41 (0.86–2.33)	1.14 (0.59–2.18)
Community level literacy				
Low proportion of illiteracy	1		1	1
High proportion of illiteracy	3.35 (2.43–4.6)		1.37 (0.95–1.98)	1.01 (0.66–1.54)
Community wealth status				
Low proportion of poor	1		1	1
High proportion of poor	2.84 (2.06–3.92)		1.08 (0.77–1.49)	0.82 (0.52–1.27)
Community media exposure				
Low proportion of non-exposed	1		1	1
High proportion of non-exposed	2.89 (2.09–3.98)		1.02 (0.71–1.48)	0.89 (0.57–1.38)
Community contraceptive use				
Low proportion of non-user	1		1	1
High proportion of non-user	2.87 (2.15–3.83)		1.61 (1.18–2.18)	1.39 (1.94–2.04)*
Region				
Tigri	1		1	1
Afar	1.46 (0.91–2.32)		0.84 (0.51–1.38)	0.32 (0.14–0.69)
Amhara	1.03 (0.6–1.77)		0.89 (0.56–1.14)	0.64 (0.37–1.09)
Oromia	1.67 (1.00–2.79)		1.57 (0.96–2.55)	1.06 (0.54–2.09)
Somali	4.1 (2.57–6.5)		2.14 (1.34–3.43)	0.89 (0.40–1.98)
Benishangul gumuz	1.93 (1.20–3.10)		1.61 (1.04–2.49)	1.25 (0.66–2.36)
SNNPR	1.77 (1.16–2.69)		1.57 (1.08–2.27)	1.39 (0.74–2.61)
Gambela	1.14 (0.62–2.10)		1.21 (0.72–2.04)	1.44 (0.66–3.14)
Harari	1.22 (0.66–2.27)		1.17 (0.70–1.95)	0.64 (0.29–1.43)
AA	0.14 (0.07–0.26)		0.34 (0.16–0.68)	0.37 (0.14–1.01)
Diredawa	1.0 (0.51–1.94)		0.96 (0.58–1.5)	0.53 (0.25–1.10)

COR, crude odds ratio; AOR, adjusted odds ratio.

* Significant (*P* < 0.05).

### Random effect

The outcomes of the random effect analysis revealed a significant correlation among observations within the same cluster, with an Intra-cluster Correlation (ICC) of 23.2%. This suggests that approximately 23% of the variation in CEB is associated with the community or cluster. The analysis further demonstrated that the full model explained 36.3% of the variation in CEB. Additionally, the Median Odds Ratio (MOR) affirmed that community-level factors significantly contributed to the variability in CEB.

In the empty model, the MOR for CEB was 2.57, indicating variation between communities (clustering), as the MOR was 2.57 times higher than the reference value (MOR = 1). Despite the continued statistical significance of clustering effects in the full models, the unexplained community variation in high number of CEB decreased to an MOR of 2.12 when considering all factors, both individual and community, in the model (refer to Table 4).

**Table 4:** Measure of variation for CEB at cluster level by multilevel logistic regression analysis, EDHS 2019.

Random effect	Null model	Model 1	Model 2	Final model
Variance	0.99	0.7	0.48	0.63
ICC%	23.2%	17.5%	12.93%	16.07%
PCV%	Reference	29.2%	51.5	36.3
MOR	2.57	2.21	1.94	2.12
Log-likelihood	−3,390.39	−2,807.59	−3,318.91	−2,804.83
AIC	6,784	5,683.07	6,673.83	5,683.65
BIC	6,798	5,822.89	6,793.66	5,929.98

ICC, intra-community correlation coefficient; PCV, proportional change in variance; AIC, Akakian information criteria; Null model model without any predictor, Model 1: a model with individual variables only, Model 2: a model with community variables only Final model- A model where both individual and community level variables were fitted simultaneously.

## Spatial distribution of children ever born

### Global and local indicator spatial autocorrelation

**Figure 3:**
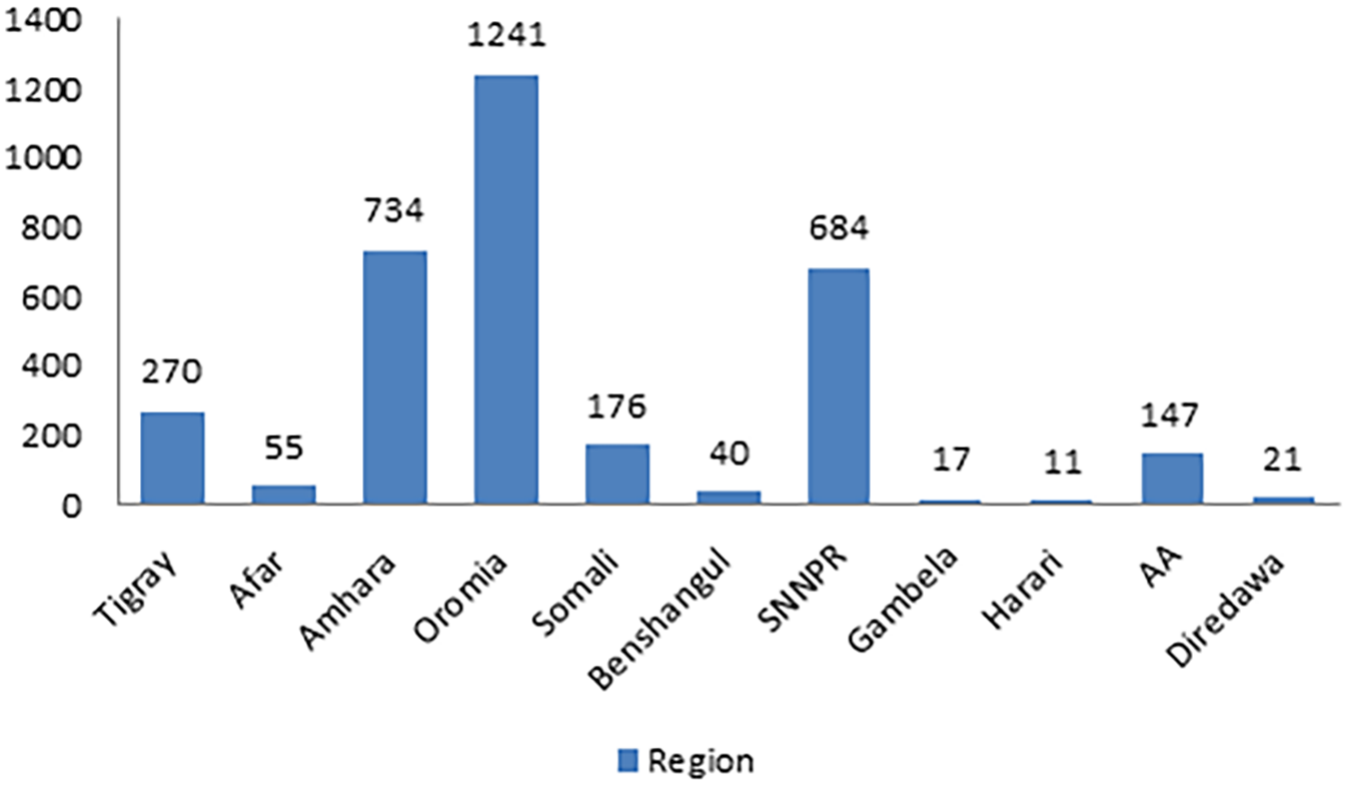
Prevalence of High Children Ever Born across different regions of Ethiopia.

**Figure 4:**
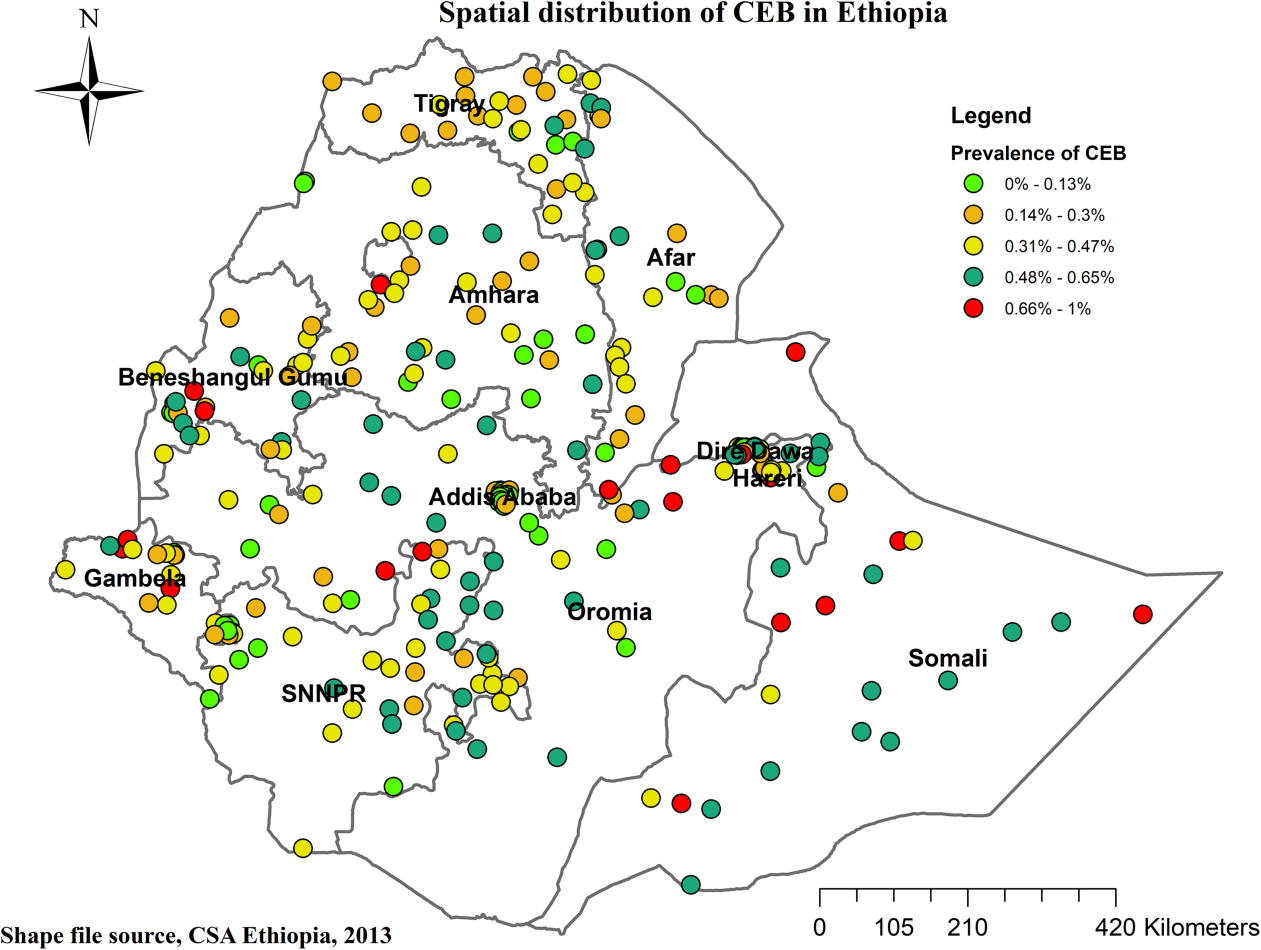
Spatial distribution of CEB in Ethiopia. 2019 MEDHS.

At the regional level, Ethiopia exhibited significant spatial variation in the prevalence of High number of CEB. The spatial distribution analysis revealed statistical significance with a Moran's index of 0.59 and a *P*-value of less than 0.000001 (refer to Figure 3). The mapping of High number of CEB across 305 clusters indicated clusters with a high prevalence located in the western parts of Benishangul, southern areas of Somali, Gambela, Harari, and Oromia Regions (refer to Figure 4).

**Figure 5:**
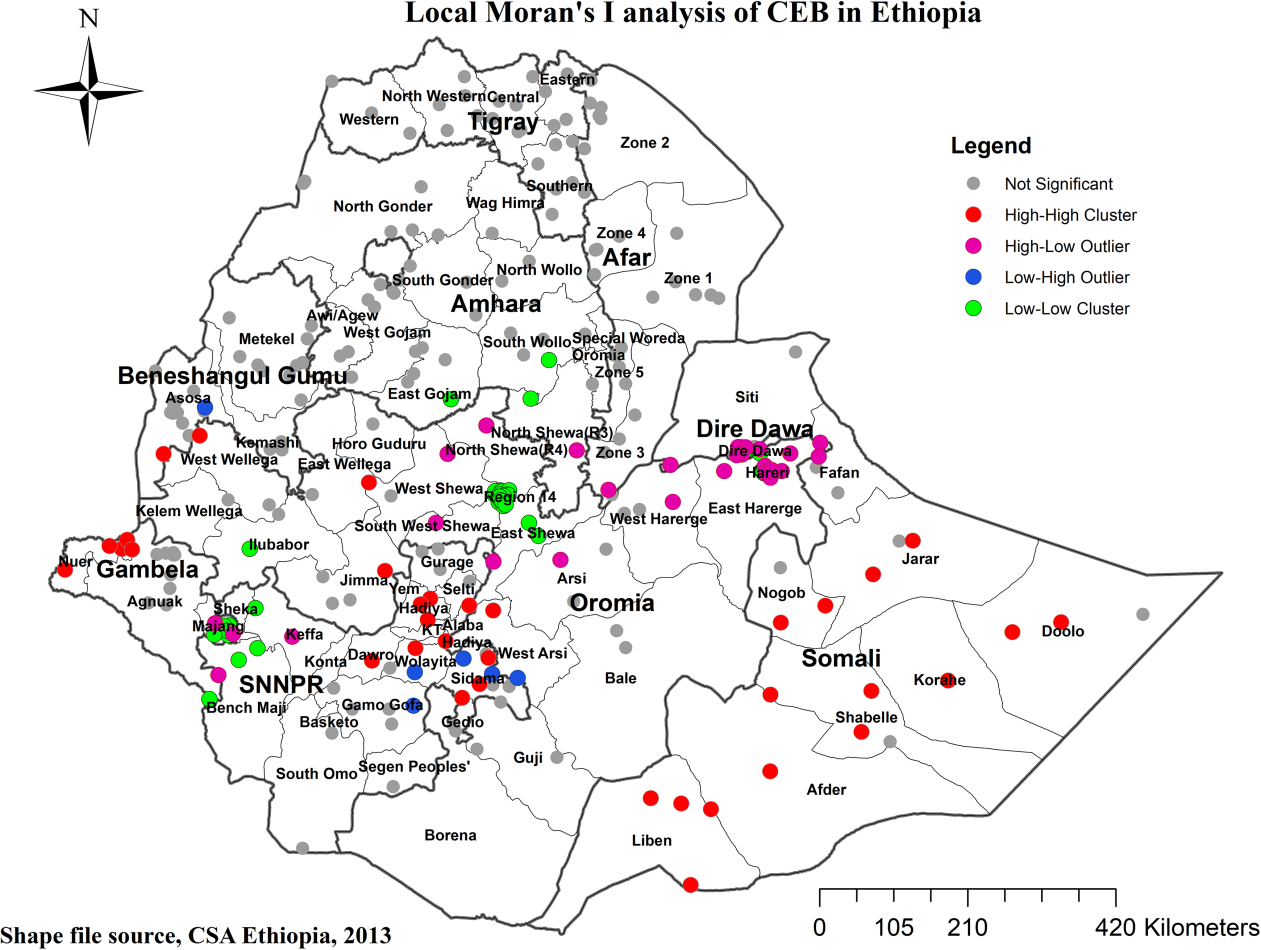
Cluster and outlier analysis of CEB in Ethiopia. 2019 MEDHS.

High high clustering (HH) denoted high prevalence of high number of CEB surrounded by high prevalence, while low low clustering (LL) indicated a low prevalence surrounded by low prevalence. However, HL and LH indicated scenarios where a high prevalence of High number of CEB was surrounded by low prevalence and vice versa. Noteworthy outliers, represented by red and blue dots, were observed in various regions, such as Somali Region, Sidama, Hadiya, Alaba, and Wellega (HH), Addis Ababa, Central Gambela, South Wollo, and East Gojam (LL), Dire Dawa, East Harari, Majiang Zone, North Shewa, West Harerge (HL), and Welayta, Gamo-Gofa, and South Assosa (LH) (refer to Figure 5).

### Hot spot analysis and interpolation

**Figure 6:**
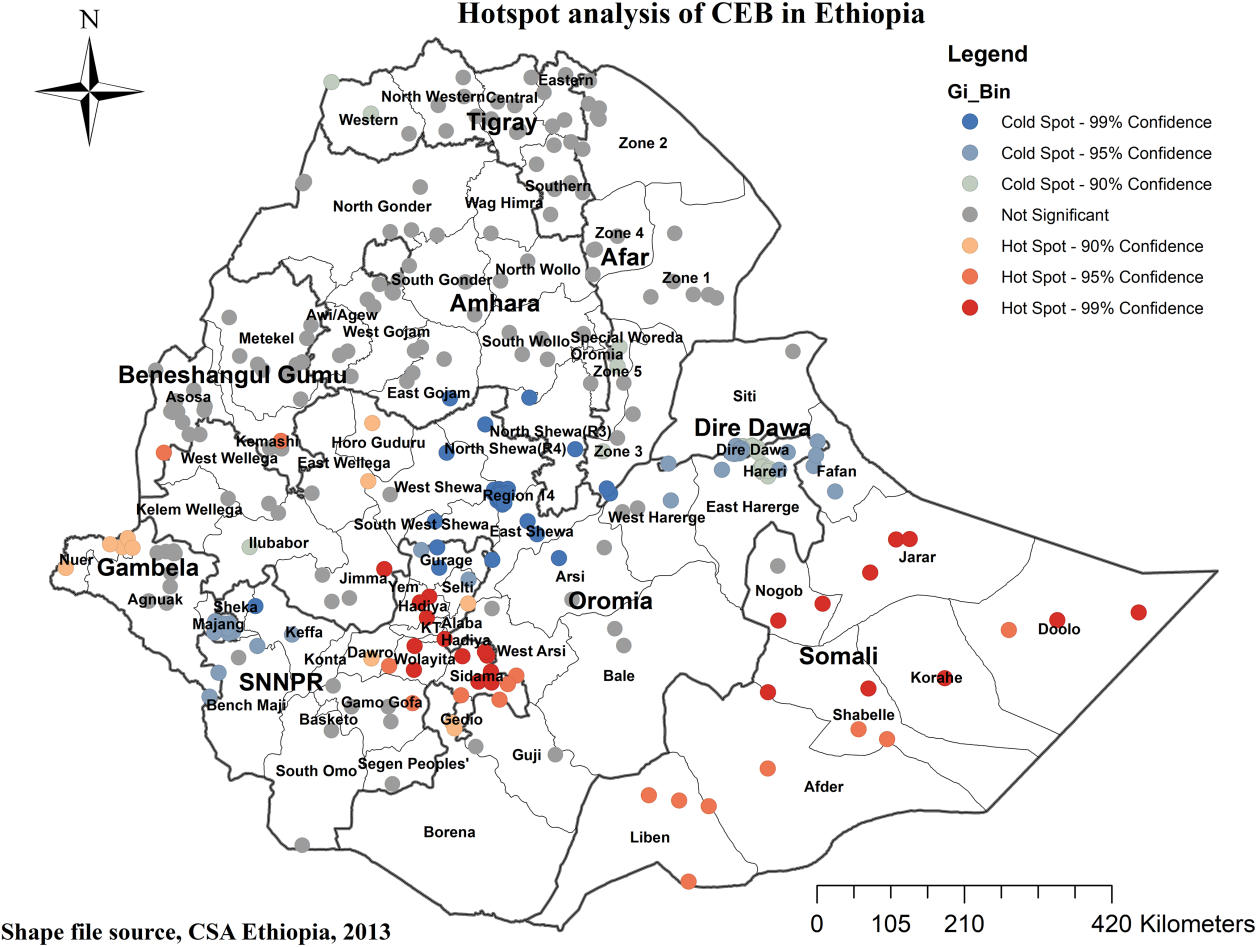
Hotspot analysis of CEB in Ethiopia. 2019 MEDHS.

The Getis-Ord Gi* statistics were employed to identify hot and cold spots, representing areas with high and low probabilities of CEB. Hot spots, marked in red, indicated regions with a high likelihood of CEB, including Eastern Somali, Hadiya, Sidama, and Welayta zones. Conversely, cold spots, marked in blue, indicated areas with a low probability, such as Dire Dawa, Harari, Southwest, North and East Shewa, and Addis Ababa (refer to Figure 6).

**Figure 7:**
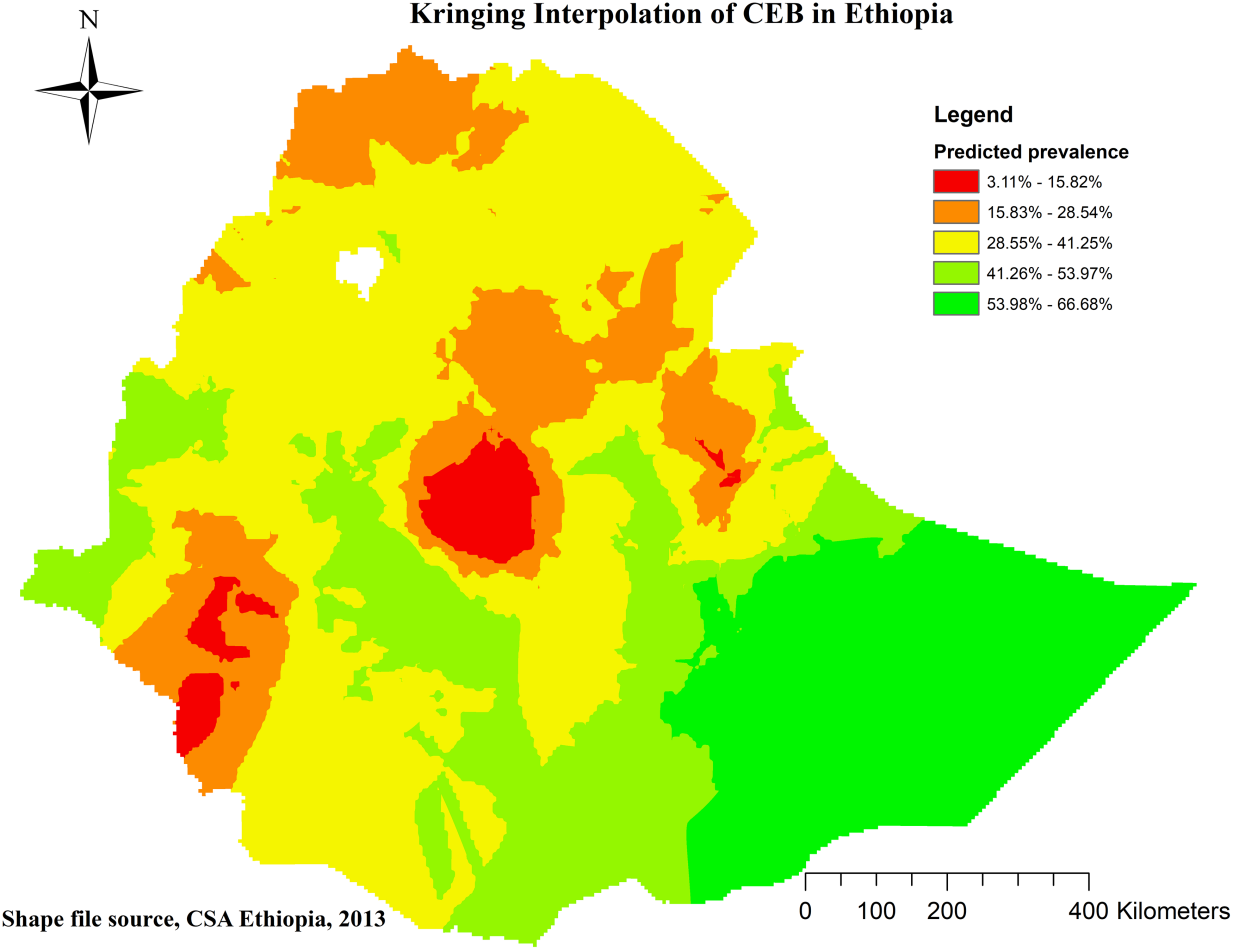
Kringing interpolation of CEB in Ethiopia. 2019 MEDHS.

Spatial kriging interpolation analysis was utilized to predict regions where data were not collected based on areas with available data. The interpolation revealed regions with a high probability of CEB, predominantly in the entirety of the Somali region, southern, eastern, and western parts of Oromia region, and the western Benishangul, Gambela, and northern South NNPR. Conversely, the administrative cities of Addis Ababa and Dire Dawa, along with their surrounding areas, were predicted to have a lower probability of CEB. The red color on the map indicates areas with a predicted high probability, while the green color represents areas with a predicted lower probability of CEB (refer to Figure 7).

## Discussion

Children Ever Born stands as one of the primary determinants shaping population dynamics, exerting a profound impact on the size, structure, and composition of a country's population. Numerous factors contribute to variations in CEB, falling into two broad categories: Individual-level factors and Community-level factors. Individual-level factors, such as women's education, age at first birth, contraceptive utilization, and marital status, play a significant role in influencing CEB in Ethiopia. Additionally, community-level factors, specifically community contraceptive utilization, also contribute significantly to the variations observed in CEB within the country. The identification of significant neighborhood clustering was crucial in understanding the spatial distribution.

According to the findings of the current study, the spatial distribution of CEB among reproductive-age women in Ethiopia exhibited clustering. Significant hotspot areas, indicating High number of CEB, were identified in Eastern Somali, Hadiya, Sidama, and Welayta zones. Conversely, significant cold spot areas, representing low CEB, were identified in Dire Dawa, Harari, Southwest, North and East Shewa, and Addis Ababa. The hotspot findings in the SNNPR region (Hadiya, Sidama, and Welayta zones) and the Somali region of Ethiopia may be explained by the high prevalence of uneducated girls in these areas—40.7% and 71.7% respectively (32). This educational disparity likely influences women's knowledge and decision-making regarding contraceptive use, leading to lower contraceptive uptake and higher fertility rates in these regions (33).

This disparity in CEB patterns could potentially be attributed to variations in socio-economic and obstetric-related factors among the study participants. For example, a majority of respondents in Addis Ababa, Dire Dawa, and Harari were reported to have higher educational levels, better access to reproductive health services like family planning, and a heightened awareness of CEB.

The educational level of women has a notable impact on the number of CEB. Specifically, women with higher education, secondary education, and primary education exhibit reductions in the number of children born by 99%, 95%, and 82%, respectively. This aligns with findings from previous research conducted in Ethiopia. A study based on the 2016 Ethiopian Demographic and Health Survey (EDHS) revealed that women who completed primary, secondary, and higher-level education experienced reductions in the number of children born by 9.7%, 47.9%, and 48%, respectively (10). Consistent findings from similar studies affirm that as the level of education increases, the desire for a high number of CEB tends to decrease (12, 13, 17, 18, 20, 22). This trend may be attributed to the fact that educated women not only possess better knowledge and skills for managing reproductive health but also tend to develop broader interests, skills, and capacities beyond parenting. Education often opens up opportunities for career development, personal growth, and social engagement, leading women (and men) to see parenting as one of many life roles rather than the central focus of their identity. This shift in perspective may naturally lead to a reduced desire for a high number of children, as individuals seek to balance various aspects of their lives (34). Additionally, higher-educated females are more inclined to delay marriage, gaining confidence to reject early marriage and resist sexual abuse (10).

The findings of this study indicate that women who entered into marriage at the age of 20 years or later were 62% less likely to have a higher number of CEB. These results align with similar findings reported in previous studies (11–13, 15, 21–23). This observation may be explained by the fact that women who initiate childbearing before the age of 20 tend to have a longer period of fertility, contributing to a higher number of CEB. Typically, these women who commence childbearing early often reside in rural areas and lack formal education, leading to limited knowledge of and access to reproductive health services, including family planning methods (13).

Contraceptive usage emerges as another influential factor in determining the number of CEB. In contrast to contraceptive non-users, individuals utilizing contraceptives were 41% less likely to have a high CEB. This finding is consistent with a study conducted in Gedo Zone, Ethiopia, supporting the notion that contraceptive use is associated with a lower likelihood of having a high number of CEB (21). The results also suggest that social norms—such as cultural attitudes toward family planning or reproductive health—can act as barriers to contraceptive use, contributing to higher fertility rates. This observation emphasizes the role of unmet needs for family planning and highlights the influence of community-level norms on women's contraceptive use (35).

The findings indicate that married women were three times more fertile compared to those who were not married or never in union. Consistent with prior research in Gondar (36) and Botswana (37), This trend highlights the entrenched social norms in Ethiopia that promote large families within marriage while stigmatizing childbirth outside of that context. Studies indicate that these cultural expectations significantly influence reproductive decisions, with contraceptive use being accepted for married women but often discouraged for single women, creating barriers to effective family planning. Despite initiatives aimed at encouraging contraceptive use among married couples, many individuals still encounter societal pressures that prioritize having many children, reinforcing the belief that personal success is closely tied to the number of children one has (38).

Community contraceptive usage plays a significant role in shaping the number of CEB. Compared to communities with a low proportion of contraceptive non-users, those with a high proportion were 39% more likely to experience a high CEB. This finding was supported by other similar studies (19, 24). A more plausible explanation is that women in these communities may be adhering to prevailing social norms that emphasize larger families and define women's identities through motherhood. These norms can limit women's reproductive choices and perpetuate high fertility rates, highlighting the need for targeted interventions that challenge these societal expectations and promote reproductive autonomy (19).

While the government endeavors to attain Sustainable Development Goals (SDGs), a considerable number of women continue to experience high fertility rates. If this issue persists or worsens, the anticipated decline in the number of children born and the reduction in maternal and child morbidity and mortality may not be realized.

## Strengths and limitations

This study employed multilevel logistic regression analysis, enabling the identification of factors at various levels and offering valuable insights for designing interventions. The results were adjusted appropriately, incorporating estimation adjustments such as weighting.

Furthermore, spatial analysis pinpointed areas with the highest concentrations of the phenomenon under investigation. Despite its strengths, it is important to acknowledge the limitations of the current study. The cross-sectional nature of the study design restricts its ability to establish a causal relationship among the associated variables and Geographically weighted regression would be more effective in identifying the specific factors that contribute to hot and cold spots.

Additionally, due to the utilization of secondary data, certain crucial variables, such as cultural beliefs and a history of abortion, were not present in the dataset, precluding their inclusion in the analysis. Finally, social desirability bias should be considered, especially concerning questions about contraceptive use.

## Conclusions

In the course of this research, it was observed that the prevalence of a high number of CEB remained elevated despite government initiatives, and its spatial distribution exhibited a non-random pattern characterized by a positive Moran's Index. Moreover, both individual and community-level factors demonstrated significant associations with a high number of CEB. Specifically, an elevated educational status of women's, a delayed age at first birth, and the utilization of contraceptives were found to have negative associations, while marital status and a high proportion of contraceptive non-users in the community exhibited positive associations with a high number of CEB in Ethiopia.

In addition to these factors, the aspect of informed choice and reproductive autonomy is crucial. Women's ability to make informed decisions regarding their reproductive health—free from coercion and with access to a range of contraceptive options can significantly influence fertility outcomes. Ensuring that women have full autonomy over their reproductive choices will further enhance the effectiveness of education and contraceptive interventions.

Therefore, it is recommended that the government focuses its interventions on reinforcing women's education, promoting a later age at first birth, encouraging the use of contraceptives, and prioritizing reproductive autonomy through informed choice to effectively address this issue.

## Data Availability

The original contributions presented in the study are included in the article/Supplementary Material, further inquiries can be directed to the corresponding author.
